# Effect of intensive prior exercise on muscle fiber activation, oxygen uptake kinetics, and oxygen uptake plateau occurrence

**DOI:** 10.1007/s00421-020-04426-1

**Published:** 2020-06-27

**Authors:** Max Niemeyer, Renate Leithäuser, Ralph Beneke

**Affiliations:** Medizin, Training und Gesundheit, Institut für Sportwissenschaft und Motologie, Philipps-Universität Marburg, Jahnstr. 12, 35037 Marburg, Germany

**Keywords:** Cardiorespiratory fitness, Priming exercise, Exercise testing, Maximum oxygen uptake, Electromyography

## Abstract

**Purpose:**

We tested the hypothesis that the described increase in oxygen uptake ($\dot{V}{\text{O}}_{{2}}$)-plateau incidence following a heavy-severe prior exercise is caused by a steeper increase in $\dot{V}{\text{O}}_{{2}}$ and muscle fiber activation in the submaximal intensity domain.

**Methods:**

Twenty-one male participants performed a standard ramp test, a $\dot{V}{\text{O}}_{{{\text{2max}}}}$ verification bout, an unprimed ramp test with an individualized ramp slope and a primed ramp test with the same ramp slope, which was preceded by an intensive exercise at 50% of the difference between gas exchange threshold and maximum workload. Muscle fiber activation was recorded from vastus lateralis, vastus medialis, and gastrocnemius medialis using a surface electromyography (EMG) device in a subgroup of 11 participants. Linear regression analyses were used to calculate the $\dot{V}{\text{O}}_{{2}}$-($\Delta \dot{V}{\text{O}}_{{2}} /\Delta P$) and EMG-(∆RMS/∆P) ramp test kinetics.

**Results:**

Twenty out of the 21 participants confirmed their $\dot{V}{\text{O}}_{{{\text{2max}}}}$ in the verification bout. The $\dot{V}{\text{O}}_{{2}}$-plateau incidence in these participants did not differ between the unprimed (*n* = 8) and primed (*n* = 7) ramp test (*p* = 0.500). The $\Delta \dot{V}{\text{O}}_{{2}} /\Delta P$ was lower in the primed compared to the unprimed ramp test (9.40 ± 0.66 vs. 10.31 ± 0.67 ml min^−1^ W^−1^, *p* < 0.001), whereas the ∆RMS/∆P did not differ between the ramp tests (0.62 ± 0.15 vs. 0.66 ± 0.14% W^−1^; *p* = 0.744).

**Conclusion:**

These findings do not support previous studies, which reported an increase in $\dot{V}{\text{O}}_{{2}}$-plateau incidence as well as steeper increases in $\dot{V}{\text{O}}_{{2}}$ and muscle fiber activation in the submaximal intensity domain following a heavy-severe prior exercise.

## Introduction

The oxygen uptake ($\dot{V}{\text{O}}_{{2}}$)-plateau is a flattening of the linear $\dot{V}{\text{O}}_{{2}}$–workload-relationship at the end of an incremental exercise test (Howley et al. 1995). It serves as an indicator of maximum oxygen uptake ($\dot{V}{\text{O}}_{{{\text{2max}}}}$), which is one of the most important measurements in exercise physiology and clinical sports medicine (Poole and Jones 2017; Hawkins et al. 2007; Levine 2008). However, even if incremental exercise has been performed with maximal effort, the observed plateau incidence is frequently less than 60% despite high levels of secondary exhaustion criteria (Lucia et al. 2006; Knaier et al. 2019b; Wood et al. 2010). It is widely believed that the occurrence of a $\dot{V}{\text{O}}_{{2}}$-plateau depends on age or aerobic fitness (Howley et al. 1995; Wagner 2000; Shephard, 2009). However, there is no consistent evidence for a higher plateau incidence in young adults compared to children and older adults (Gürsel et al. 2004; Peyer et al. 2011; Edvardsen et al. 2014) or in trained compared to untrained participants (Lucia et al. 2006; Doherty et al. 2003; Day et al. 2003; Edvardsen et al. 2014; Wood et al. 2010). Instead, it has been suggested that a high anaerobic capacity increases the chance that a $\dot{V}{\text{O}}_{{2}}$-plateau occurs (Gordon et al. 2011).

In a subsequent study, Gordon et al. (2012) found an increase in the plateau incidence from 50 to 100% following a prior exercise at 50% of the difference between the workload at gas exchange threshold (*P*_GET_) and maximum workload (*P*_max_). They interpreted this finding as evidence for the hypothesis that the $\dot{V}{\text{O}}_{{2}}$-plateau depends on anaerobic capacity. This interpretation is based on studies that reported a slower accumulation of anaerobic metabolites following a heavy or severe priming bout (Bangsbo et al. 2001; Layec et al. 2009), suggesting a potential sparing of anaerobic capacity. Because of a rather slow time-dependent behavior of $\dot{V}{\text{O}}_{{2}}$, there is a continuous accumulation of $\dot{V}{\text{O}}_{{2}}$ deficit during incremental exercise tests (Niemeyer et al. 2019; Pouilly and Busso. 2008), which lead to an accumulation of anaerobic metabolites up to the individual tolerable limit (Raymer et al. 2007). Therefore, a priming-induced slower accumulation of anaerobic metabolites (i.e., a slower reduction in anaerobic capacity) should result in higher *P*_max_, as demonstrated by Raymer et al. 2007. Since for the diagnoses of a $\dot{V}{\text{O}}_{{2}}$-plateau, $\dot{V}{\text{O}}_{{{\text{2max}}}}$ must be sustained despite rather large increases in workload (Niemeyer et al. 2020), a higher *P*_max_ at given $\dot{V}{\text{O}}_{{{\text{2max}}}}$ should in turn increase the chance that a plateau occurs. However, Gordon et al, (2012) did not find corresponding differences in *P*_max_ between primed and the unprimed ramp tests. Therefore, it seems to be unlikely that the observed increase in plateau incidence is caused by an increase or sparing of anaerobic capacity. Additionally, Gordon et al. (2012) used a $\dot{V}{\text{O}}_{{2}}$-plateau definition that results in a high incidence of a false plateau diagnosis (Niemeyer et al. 2020), which questions their findings considerably.

As recently demonstrated, participants with a $\dot{V}{\text{O}}_{{2}}$-plateau had lower time constants of $\dot{V}{\text{O}}_{{2}}$ square wave kinetics, but also steeper slopes of the $\dot{V}{\text{O}}_{{2}}$–workload-relationship ($\Delta \dot{V}{\text{O}}_{{2}} /\Delta P$) in incremental ramp tests (Niemeyer et al. 2019). It is well known that the overall $\dot{V}{\text{O}}_{{2}}$ response kinetics is accelerated after priming exercise bouts in the heavy or severe intensity domain (Bailey et al. 2009; Burnley et al. 2000, 2006). Since the ramp test $\dot{V}{\text{O}}_{{2}}$ response is modulated by the time constant and gain of $\dot{V}{\text{O}}_{{2}}$ overall kinetics, but also by the intensity-dependent increase in these parameters (Wilcox et al. 2016) in incremental ramp tests this led to an increase in $\Delta \dot{V}{\text{O}}_{{2}} /\Delta P$ at workloads below and/or above *P*_GET_ (Jones and Carter 2004; Boone et al. 2012). At a given $\dot{V}{\text{O}}_{{{\text{2max}}}}$, a higher $\Delta \dot{V}{\text{O}}_{{2}} /\Delta P$ results in faster attainment of $\dot{V}{\text{O}}_{{{\text{2max}}}}$ and therefore potentially contributes to the development of a $\dot{V}{\text{O}}_{{2}}$-plateau despite a similar *P*_max_ (Niemeyer et al. 2019). Thus, it seems to be likely that the potential for an increase in the $\dot{V}{\text{O}}_{{2}}$-plateau incidence in the primed condition is caused by an increase in $\Delta \dot{V}{\text{O}}_{{2}} /\Delta P$ rather than an increase or sparing of anaerobic capacity. This alternative explanation would support the hypothesis that the $\dot{V}{\text{O}}_{{2}}$-plateau is affected by $\dot{V}{\text{O}}_{{2}}$-kinetics (Niemeyer et al. 2019). However, no such analysis has previously been reported.

Boone et al. (2012) showed that the increase in $\Delta \dot{V}{\text{O}}_{{2}} /\Delta P$ following priming exercise is accompanied by a steeper increase in the electromyography amplitude signal per increase in workload (∆RMS/∆P). This indicates that the prior exercise-induced increase in $\Delta \dot{V}{\text{O}}_{{2}} /\Delta P$ is probably caused by elevated muscle fiber activation. Taken together therefore, we tested the hypothesis that the increase in $\dot{V}{\text{O}}_{{2}}$-plateau incidence following a prior exercise is caused by a higher ∆RMS/∆P and $\Delta \dot{V}{\text{O}}_{{2}} /\Delta P$ at intensities below $\dot{V}{\text{O}}_{{{\text{2max}}}}$.

## Methods

We recruited 21 male students (age 25.2 ± 2.7 years; height 180.7 ± 5.4 cm; body mass 76.6 ± 6.1 kg), who were physically active but not cycling-specifically trained. The sample size was previously calculated using a power analysis for a one-tailed McNemar test. Based on the results of Gordon et al. (2012), we assumed that maximal 10% of the participants do not show a plateau in the primed ramp test and set the power at 90% requiring a sample size of *n* = 13. To fulfill the minimum required counts per cell of the McNemar test (*n* ≥ 5), the sample size was increased to 21. All participants gave their written informed consent to participate in this study after detailed information about the potential risks of their participation. The study was approved by the ethics committee of Philipps-University Marburg, Department of Education Science (FB-21-AZ4-11-16) and conformed to the Declaration of Helsinki.

### Study design

We used a randomized cross-over design to test the hypothesis. The study consisted of a series of three tests on a cycle ergometer (Cyclus-2 RBM elektronik-automation GmbH, Germany), which were separated by at least 48 h. On the first testing day, the participants performed a standard incremental ramp test up to exhaustion with a workload increment of 30 W min^−1^. On testing days two and three, the participants performed either an unprimed or a primed ramp test with an individualized incremental rate in randomized order, respectively. The incremental rate (*S*) was previously calculated from the maximal workload (*P*_max_) of the standard ramp test of the first testing day to allow a comparable time to exhaustion for all participants of ~ 10 min (Eq. 1).

1 $$S = \frac{{P_{\max } - 50}}{10}.$$

All ramp tests started with three minutes baseline-cycling at 50 W. The primed ramp test was preceded by a three-step square-wave exercise with step durations of 6 min each. The workloads of the three steps were set at 50 W (step 1), 90% of the workload at the gas exchange threshold (*P*_GET_) (step 2) and 50% of the difference between *P*_GET_ and *P*_max_ (step 3). Between step 3 and the ramp test, a 6-min active recovery at a workload of 50 W was performed. The unprimed ramp test was followed by a 12-min active recovery at 50 W and a subsequent $\dot{V}{\text{O}}_{{{\text{2max}}}}$ verification bout. In accordance with other studies and to allow a minimum time to exhaustion of ≥ 2 min, the workload of the verification bout was set at 90% of *P*_max_ (Day et al. 2004; Sedgeman et al. 2013). The pedalling rate during the tests was set at 80 rpm, and the temperature was kept constant at 20 °C by air condition. To avoid potential effects of diurnal variation on $\dot{V}{\text{O}}_{{{\text{2max}}}}$ (Knaier et al. 2019a), all tests were performed at the same time of day (± 1 h).

### Measurements

Expired air was continuously measured throughout the entire tests using a breath-by-breath device (MetaMax 3B, Cortex Biophysik GmbH, Germany). Before each test, the device was calibrated with a mixture of known gases (15% O_2_, 5% CO_2_ and 80% N) and a 3-l syringe.

Muscle fiber activations of vastus lateralis (VL), vastus medialis (VM), and gastrocnemius medialis (GM) were measured bilateral in a subgroup of 11 participants with a surface electromyography system (TeleMyo 16-EMG DTS Noraxon USA Inc.; Scottsdale, Arizona, USA). The EMG signal was recorded at a sampling rate of 3000 Hz using Ag/AgCl-electrodes with a 10 mm diameter. The electrodes were attached to the skin at a constant inter-electrode distance of 20 mm and located according to the SENIAM-guidelines (Hermens et al. 1999). Previously, the skin was carefully shaved and abraded using abrasive paper and alcohol to keep the inter-electrode impedance below 2000 Ω. To enable the same location of electrodes in each test, the position was tagged with an indelible marker.

Blood lactate concentrations (BLC) were determined from 20-µl capillary blood samples. The samples were collected from hyperaemic earlobes at rest, during the final 30 s of the 50 W baseline-cycling before the start of the ramp test, at the end of the ramp test as well as 1, 3, and 5 min after ramp test termination. Subsequently, the samples were analyzed using an enzymatic-amperometric analyzer (Biosen C-Line, EKF-diagnostic GmbH, Germany). Maximal rating of perceived exertion (RPE_max_) was assessed at the end of the ramp test with Borg scale ranging from 6 to 20.

### Data analyses and statistics

In the first step, $\dot{V}{\text{O}}_{{2}}$ was filtered by removing all values that differ more than three standard deviations from the local mean (Lamarra et al. 1987; Keier et al. 2014). $\dot{V}{\text{O}}_{{{\text{2max}}}}$, maximal carbon dioxide production ($\dot{V}{\text{CO}}_{{{\text{2max}}}}$), and maximal respiratory exchange ratio (RER_max_) were calculated as the mean of the final 30 s of the ramp and verification test. Based on the test–retest reliability of $\dot{V}{\text{O}}_{{{\text{2max}}}}$ (Katch et al. 1990; Knaier et al. 2019a), participants with a $\dot{V}{\text{O}}_{{2}}$ in the verification bout more than 5% higher compared to the unprimed ramp test were excluded from the subsequent analyses. The $\dot{V}{\text{O}}_{{2}}$-plateau was determined from the slope of the $\dot{V}{\text{O}}_{{2}}$–workload-relationship of the final 50 W ($\Delta \dot{V}{\text{O}}_{{{\text{2max}}}}$) using linear regression analyses. Based on previous recommendations (Niemeyer et al. 2020), a plateau was accepted if the slope was less than 5.0 ml min^−1^ W^−1^, which represents approximately half of the expected slope in the submaximal intensity domain (Boone and Bourgois 2012).

Linear regression analyses were also used to calculate the $\Delta \dot{V}{\text{O}}_{{2}} /\Delta P$ (Eq. 2), where the slope (m) of the $\dot{V}{\text{O}}_{{2}}$–workload-relationship represents the $\Delta \dot{V}{\text{O}}_{{2}} /\Delta P$. Furthermore, *P* is defined as the workload, and b as the intercept.

2 $$\dot{V}O_{2} = m \cdot P + b.$$

In accordance with Jones and Carter (2004), we calculated the $\Delta \dot{V}{\text{O}}_{{2}} /\Delta P$ in three submaximal intensity domains: from one minute into the ramp test up to *P*_GET_ (S1), from *P*_GET_ up to two minutes before ramp test termination (S2), and from one minute into the ramp test up to 2 min before ramp test termination (ST). *P*_GET_ was previously determined using the *V*-slope method (Beaver et al. 1986). The mean response time of the $\dot{V}{\text{O}}_{{2}}$ ramp test response (MRT) was calculated from the interception point of the $\dot{V}{\text{O}}_{{2}}$–workload-relationship below *P*_GET_ (S1) and a horizontal line crossing the $\dot{V}{\text{O}}_{{2}}$ of 50 W baseline-cycling, which precedes the ramp test (Eq. 3):

3 $${\text{MRT}} = \frac{{\dot{V}O_{{2{\text{BSL}}}} - b\_S1}}{{\Delta \dot{V}O_{2} /\Delta P\_S1 \cdot S}} - \frac{50}{S},$$

where $\dot{V}{\text{O}}_{{{\text{2BSL}}}}$ was previously calculated as the mean $\dot{V}{\text{O}}_{{2}}$ of the last 60 s of the 50 W baseline cycling and *S* was the ramp slope with the unit W s^−1^. $\Delta \dot{V}{\text{O}}_{{2}} /\Delta P\_S1$ and *b*_*S*1 were defined as slope and the intercept of the $\dot{V}{\text{O}}_{{2}}$–workload-relationship below *P*_GET_ (S1).

The raw EMG signal was bandpass filtered between 10 and 500 Hz, rectified and smoothed using a moving root mean square (RMS) of 100 ms. Subsequently, the RMS was averaged over 10 s intervals and time-aligned to the beginning of the ramp test. Then, we averaged the RMS of both legs separately for every muscle (VL; VM and GM) as well as for all muscles together (all). Finally, the ramp test RMS was normalized to the mean RMS of the last minute of the 50 W-baseline cycling, which precedes the ramp tests (Mirka 1991; Boone et al. 2012). The RMS–workload relationship was quantified by calculating the ∆RMS/∆P over the same workload ranges as previously performed for the $\Delta \dot{V}{\text{O}}_{{2}} /\Delta P$ using linear regression analyses.

**Fig. 1:**
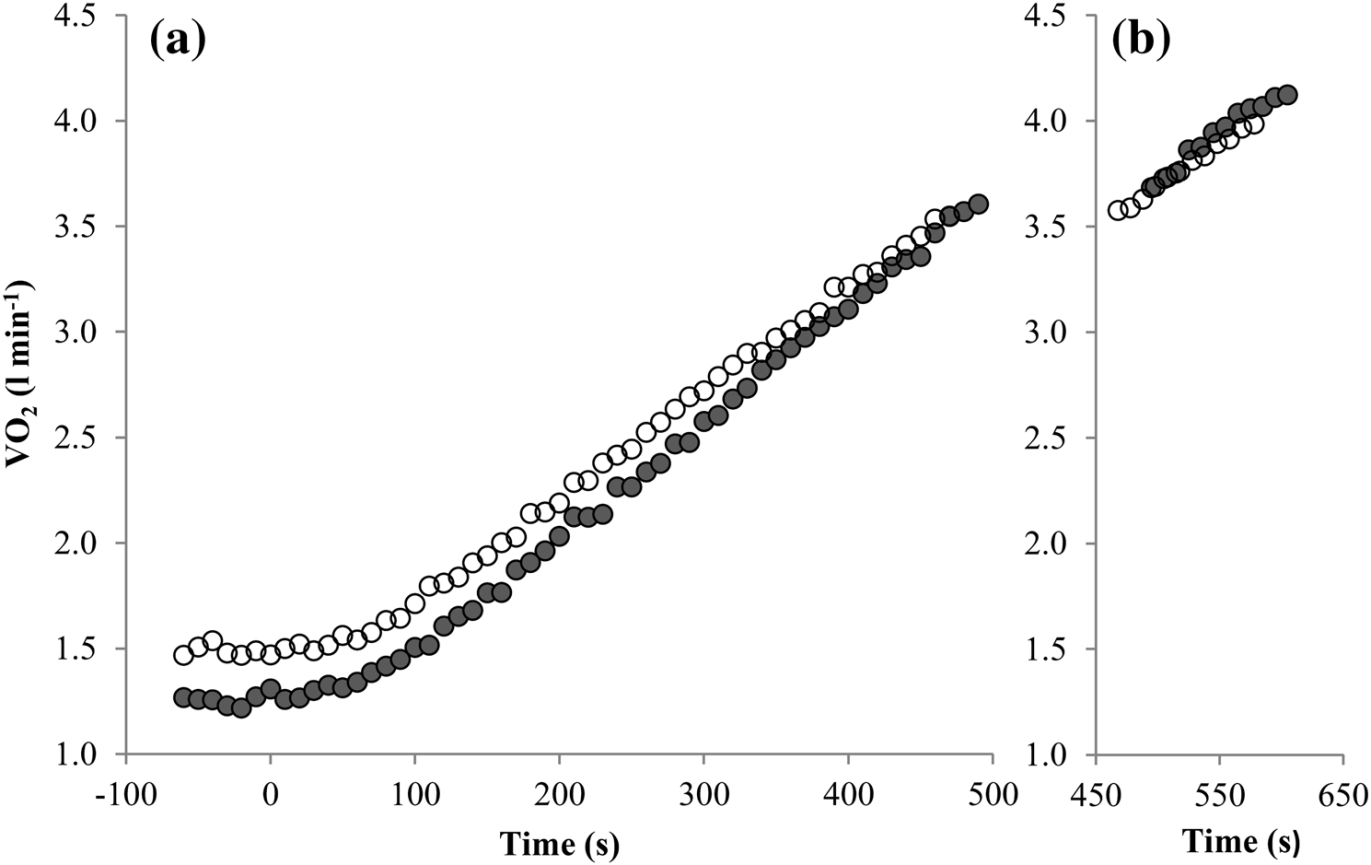
Mean group (*n* = 20) $\dot{V}{\text{O}}_{{2}}$-kinetics of the unprimed (closed circle) and primed (open circle) ramp test; **a** up to 120 s before ramp test termination; **b** during the final 120 s

**Fig. 2:**
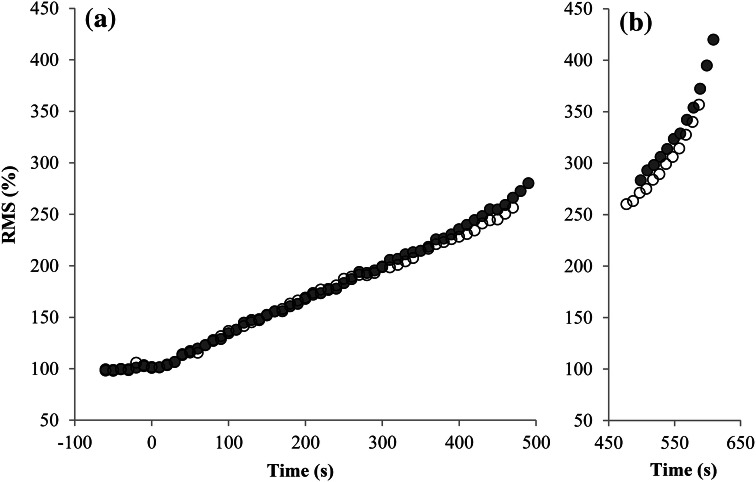
Mean subgroup (*n* = 11) EMG-kinetics (average of all muscles) of the unprimed (closed circle) and primed (open circle) ramp test; **a** up to 120 s before ramp test termination; **b** during the final 120 s

Descriptive data are presented as mean ± SD. We compared the plateau incidences in the unprimed and primed ramp tests using a one-tailed McNemar test and calculated the effect size Cohen's *g*. All other maximal exercise data of both ramp tests were compared with *t* tests for paired samples and the corresponding effect size Cohen's d. Analyses of variance were used to compare the $\Delta \dot{V}{\text{O}}_{{2}} /\Delta P$ and ∆RMS/∆P between the conditions. For this purpose, the separate intensity regions of the $\dot{V}{\text{O}}_{{2}}$ and RMS-workload slopes (*S*1, *S*2 and ST) and the prior exercise (unprimed vs. primed) were defined as two independent within-subject factors. *η*^2^ was calculated as the corresponding effect size. Post hoc comparisons were performed using *t* tests for paired samples and Bonferroni adjustments. To represent the mean $\dot{V}{\text{O}}_{{2}}$ and RMS responses in Figs. 1 and 2, we calculated the mean values in 10 s intervals and averaged the values of all participants. To enable a similar number of cases despite varying times to exhaustion, we averaged the 10 s intervals up to the average time to exhaustion minus 2 min (submaximal intensity domain) as well as the final 2 min of every single participant (maximal intensity domain).

## Results

One participant showed a more than 5% higher $\dot{V}{\text{O}}_{{{\text{2max}}}}$ in the verification test compared with the unprimed ramp test and was excluded from the subsequent analyses. As shown in Table 1, the plateau incidence (*p* = 0.500; *g* = 0.00) as well as the corresponding $\Delta \dot{V}{\text{O}}_{{{\text{2max}}}}$ (*p* = 0.962; *d* = 0.01) did not differ between the unprimed and primed ramp tests. Five out of the eight participants that had a plateau in the unprimed ramp test showed a plateau in the primed ramp test also. Three participants had a plateau in the unprimed but not in the primed ramp test. Two participants showed no plateau in the unprimed but in the primed ramp test. $\dot{V}{\text{O}}_{{{\text{2max}}}}$ (*p* = 0.032; *d* = 0.52), $\dot{V}{\text{CO}}_{{{\text{2max}}}}$ (*p* < 0.001; *d* = 1.65), *P*_max_ (*p* = 0.001; *d* = 0.86), RER_max_ (*p* < 0.001; *d* = 1.30) and BLC_max_ (*p* = 0.006; *d* = 0.69) were significantly lower in the primed ramp test. Only BLC_BSL_ (*p* < 0.001; *d* = 2.48) and RPE_max_ (*p* = 0.014; *d* = 0.60) were higher in the primed than in the unprimed ramp test.

**Table 1:** Maximal and submaximal exercise data of the unprimed and primed ramp test (*n* = 20)

	Unprimed	Primed
Plateau incidence (*n* (%))	8 (40%)	7 (35%)
$\Delta \dot{V}{\text{O}}_{{{\text{2max}}}}$ (ml·min^−1^·W^−1^)	6.90 ± 4.30	6.84 ± 5.07
$\dot{V}{\text{O}}_{{{\text{2max}}}}$ (l·min^−1^)	4.12 ± 0.49	3.97 ± 0.52*
$\dot{V}{\text{CO}}_{{{\text{2max}}}}$ (l·min^−1^)	5.14 ± 0.64	4.61 ± 0.71*
*P*_max_ (W)	371.0 ± 51.4	358.2 ± 56.6*
RER_max_	1.25 ± 0.05	1.16 ± 0.06*
BLC_max_ (mmol·l^−1^)	14.57 ± 1.70	13.27 ± 1.36*
BLC_BSL_ (mmol l^−1^)	1.00 ± 0.22	7.63 ± 2.67*
RPE_max_	18.6 ± 1.4	19.2 ± 1.3*

Data are Mean ± SD

$\Delta \dot{V}O_{2max}$ increase in $\dot{V}{\text{O}}_{2}$ within the last 50 W, $\dot{V}O_{2max}$ maximum oxygen uptake, $\dot{V}CO_{2max}$ maximum carbon dioxide production, *P*_*max*_ maximum workload, *RER*_*max*_ maximal respiratory exchange ratio, *BLC*_*max*_ maximal blood lactate concentration, *BLC*_*BSL*_ blood lactate concentration at 50 W-baseline cycling before the corresponding ramp tests, *RPE*_*max*_ maximal rating of perceived exertion

*Significantly different from the unprimed ramp test

### $\dot{V}{\text{O}}_{{2}}$-kinetics

The $\dot{V}{\text{O}}_{{2}}$-kinetics of the unprimed and primed ramp tests is displayed in Fig. 1 and Table 2. The $\dot{V}{\text{O}}_{{2}}$ at the end of the 50 W baseline cycling was significantly higher before the primed than in the unprimed ramp test (*p* < 0.001; *d* = 1.73). No significant difference between the conditions was evident with respect to the MRT (*p* = 0.902; *d* = 0.03). The ANOVA revealed no effect of the exercise intensity domain on the $\Delta \dot{V}{\text{O}}_{{2}} /\Delta P$ (*F* (1.09, 20.79) = 1.600; *p* = 0.222; *η*^2^ = 0.08), which indicates that $\Delta \dot{V}{\text{O}}_{{2}} /\Delta P$ did not differ over the three distinct intensity regions (S1, S2 and ST) In contrast, the $\Delta \dot{V}{\text{O}}_{{2}} /\Delta P$ was significantly affected by the prior exercise (*F* (1, 19) = 21.66; *p* < 0.001; *η*^2^ = 0.53). Subsequent pairwise comparisons revealed significantly lower $\Delta \dot{V}{\text{O}}_{{2}} /\Delta P$ in the primed ramp test in the S2 (*p* < 0.001; *d* = 1.24) and ST (*p* < 0.001; *d* = 1.48) intensity domains.

**Table 2:** $\dot{V}{\text{O}}_{{2}}$-kinetics of the unprimed and primed ramp test (*n* = 20)

	Unprimed	Primed
$\dot{V}{\text{O}}_{{{\text{2BSL}}}}$ (l·min^−1^)	1.26 ± 0.10	1.49 ± 0.16*
MRT (s)	45.5 ± 13.0	46.1 ± 20.0
$\Delta \dot{V}{\text{O}}_{{2}} /\Delta P$_S1 (ml·min^−1^ W^−1^)	9.56 ± 0.89	9.36 ± 2.28
$\Delta \dot{V}{\text{O}}_{{2}} /\Delta P$_S2 (ml·min^−1^ W^−1^)	10.41 ± 0.69	9.21 ± 0.79*
$\Delta \dot{V}{\text{O}}_{{2}} /\Delta P$_ST (ml·min^−1^ W^−1^)	10.31 ± 0.67	9.40 ± 0.66*

Data are Mean ± SD

$\dot{V}O_{2BSL}$ oxygen uptake at 50 W-baseline cycling before the corresponding ramp tests, *MRT* mean response time of ramp test $\dot{V}{\text{O}}_{{2}}$-kinetics, $\Delta \dot{V}O_{2} /\Delta P$ slope of the linear oxygen uptake–workload-relationship, *S1* intensity between the second minute of ramp test and *P*_GET_, *S2* intensity between *P*_GET_ and 2 min before ramp test termination, *ST* intensity between the second minute of ramp test and 2 min before ramp test termination

*Significantly different from the unprimed ramp test

### EMG-kinetics

Figure 2 and Table 3 show the EMG-kinetics of the unprimed and primed ramp tests. RMS_BSL_ of the three recorded muscles appeared to be unaffected by the prior exercise (VL: *p* = 0.052; *d* = 0.66; VM: *p* = 0.280; *d* = 0.34; GM: *p* = 0.317; *d* = 0.32; all: *p* = 0.243; *d* = 0.37). Also, ∆RMS/∆P of the three recorded muscles did not differ between the primed and unprimed ramp tests (VL: *F* (1.00, 10.00) = 3.796; *p* = 0.080; *η*^2^ = 0.28; VM: *F* (1.00, 10.00) = 0.101; *p* = 0.758; *η*^2^ = 0.01; GM: *F* (1.00, 10.00) = 1.421; *p* = 0.261; *η*^2^ = 0.12; all: *F* (1.00, 10.00) = 0.113; *p* = 0.744; *η*^2^ = 0.01). Furthermore, ∆RMS/∆P did not differ over the three exercise intensity domains (VL: *F* (1.05, 10.52) = 1.604; *p* = 0.234; *η*^2^ = 0.14; VM: *F* (1.11, 11.14) = 0.179; *p* = 0.707; *η*^2^ = 0.02; GM: *F* (1.29, 12.86) = 1.161; *p* = 0.318; *η*^2^ = 0.10; all: *F* (1.09, 10.87) = 0.220; *p* = 0.668; *η*^2^ = 0.02) and there were no interaction effects between the exercise intensity domains and the prior exercise (VL: *F* (1.07, 10.67) = 1.561; *p* = 0.240; *η*^2^ = 0.14; VM: *F* (1.08, 10.80) = 0.392; *p* = 0.560; *η*^2^ = 0.04; GM: *F* (1.02; 10.17) = 1.013; *p* = 0.339; *η*^2^ = 0.09; all: *F* (1.09, 10.89) = 1.766; *p* = 0.213; *η*^2^ = 0.15).

**Table 3:** EMG-kinetics of the unprimed and primed ramp test (*n* = 11)

Muscle	Measurement	Unprimed	Primed
Vastus lateralis	RMS_BSL_ (μV)	32.4 ± 8.2	34.8 ± 9.5
	∆RMS/∆*P*_*S*1 (% W^−1^)	0.94 ± 0.33	0.93 ± 0.33
	∆RMS/∆*P*_*S*2 (% W^−1^)	1.15 ± 0.41	0.97 ± 0.37
	∆RMS/∆P_ST (% W^−1^)	1.08 ± 0.34	0.94 ± 0.28
Vastus medialis	RMS_BSL_ (μV)	50.2 ± 14.0	53.0 ± 15.6
	∆RMS/∆*P*_*S*1 (% W^−1^)	1.13 ± 0.51	1.18 ± 0.46
	∆RMS/∆*P*_*S*2 (% W^−1^)	1.14 ± 0.40	1.06 ± 0.45
	∆RMS/∆P_ST (% W^−1^)	1.15 ± 0.35	1.09 ± 0.41
Gastrocnemius medialis	RMS_BSL_ (μV)	74.5 ± 25.4	78.7 ± 22.6
	∆RMS/∆*P*_*S*1 (% W^−1^)	0.16 ± 0.13	0.19 ± 0.19
	∆RMS/∆*P*_*S*2 (% W^−1^)	0.26 ± 0.19	0.19 ± 0.15
	∆RMS/∆P_ST (% W^−1^)	0.21 ± 0.14	0.18 ± 0.12
All muscles	RMS_BSL_ (μV)	52.4 ± 10.6	54.6 ± 14.5
	∆RMS/∆*P*_*S*1 (% W^−1^)	0.61 ± 0.20	0.66 ± 0.16
	∆RMS/∆*P*_*S*2 (% W^−1^)	0.69 ± 0.22	0.63 ± 0.20
	∆RMS/∆P_ST (% W^−1^)	0.66 ± 0.14	0.62 ± 0.15

Data are Mean ± SD

*RMS*_*BSL*_ root mean square of the EMG-signal at the final minute of 50 W-baseline cycling before the corresponding ramp tests, *∆RMS/∆P* slope of the root mean square–workload-relationship, *S1* intensity between the second minute of ramp test and *P*_GET_, *S2* intensity between *P*_GET_ and 2 min before ramp test termination, *ST* intensity between the second minute of ramp test and 2 min before ramp test termination

## Discussion

The main finding of our study is that a heavy-severe prior exercise did not change the $\dot{V}{\text{O}}_{{2}}$-plateau incidence. Furthermore, we did not find an increase in ∆RMS/∆P and even a reduction in $\Delta \dot{V}{\text{O}}_{{2}} /\Delta P$ in the S2 and ST intensity domains. The outcomes of our study are therefore contrary to our hypothesis as well as previous findings (Jones and Carter 2004; Boone et al. 2012; Gordon et al. 2012).

### Effect of prior exercise on the $\dot{V}{\text{O}}_{{2}}$-plateau incidence

Gordon et al. (2012) described in a group of 12 cyclists a significant increase in the plateau incidence from 50 to 100% after a prior exercise at 50% of the difference between *P*_GET_ and *P*_max_. Furthermore, they found a trend towards an increase from 50 to 82% after a prior exercise at 80% of the difference between *P*_GET_ and *P*_max_. Although we used a comparable prior exercise, which also comprised a 6-min exercise bout at 50% of the difference between *P*_GET_ and *P*_max_ as well as a subsequent 6-min active recovery, the plateau incidence was mostly unaffected in our study.

In addition to this outcome, we also found a significant reduction in $\dot{V}{\text{O}}_{{{\text{2max}}}}$ and *P*_max_ in the primed compared with the unprimed ramp test. According to previous prior exercise studies, this indicates that the recovery between the prior exercise and the subsequent ramp test was too short, which results in an impaired anaerobic capacity and a reduced exercise tolerance (Ferguson et al. 2007; Bailey et al. 2009; Wittekind et al. 2012). Since the occurrence of a $\dot{V}{\text{O}}_{{2}}$-plateau has been related to anaerobic capacity (Gordon et al. 2011), the absence of an increase in the plateau incidence may be caused by a too fatiguing prior exercise or insufficient recovery. However, even in the participants with a mostly similar *P*_max_ (± 10 W) in the unprimed and primed ramp test (*n* = 9) we did not find an increase in the plateau incidence (*n* = 5 and 4 in the unprimed and primed ramp test, respectively). Therefore, an insufficient recovery of anaerobic capacity and the resulting reduction in *P*_max_ may not be the sole cause for the absence of an increase in the plateau incidence.

Another potential reason for the divergent findings may be the procedure of $\dot{V}{\text{O}}_{{2}}$-plateau determination. We accepted a plateau when the $\Delta \dot{V}{\text{O}}_{{2}} /\Delta P$ of the final 50 W was less than 5.0 ml min^−1^ W^−1^. As recently shown (Niemeyer et al. 2020), this definition enables to detect a plateau with a risk of false plateau diagnoses below 5%. Gordon et al. (2012) determined the $\dot{V}{\text{O}}_{{2}}$-plateau from the difference between the last and next-to-last 30 s of a ramp test with an incremental rate of 30 W min^−1^. They accepted a plateau when the difference was less than 2.1 ml min^−1^ kg^−1^. Since the mean workload of two consecutive 30 s intervals differs by about 15 W, the expected mean increase in $\dot{V}{\text{O}}_{{2}}$ in the submaximal intensity domain is ~ 150 ml min^−1^ (assuming a $\Delta \dot{V}{\text{O}}_{{2}} /\Delta P$ of 10 ml min^−1^ W^−1^). Because the average body mass in the study by Gordon et al. (2012) was 69 kg, their mean cut-off was in fact set at 145 ml min^−1^, which is only 5 ml min^−1^ below the expected increase in $\dot{V}{\text{O}}_{{2}}$, if no plateau occurs. If the plateau definition of Gordon et al. (2012) is applied to our data, the plateau incidence in the unprimed and primed ramp tests increases to 80 and 65%, respectively. It is therefore very likely that the findings of Gordon et al. (2012) are affected by a high frequency of false-positive plateau diagnoses (Niemeyer et al. 2020).

### Effect of prior exercise on $\Delta \dot{V}{\text{O}}_{{2}} /\Delta P$

Contrary to our hypothesis, we found a comparable $\Delta \dot{V}{\text{O}}_{{2}} /\Delta P$ in the *S*1 and even a reduction in the *S*2 and ST intensity domains, as shown in Fig. 1 and Table 2. Previously, an increase in $\Delta \dot{V}{\text{O}}_{{2}} /\Delta P$ after a prior exercise has been described. However, the findings are inconsistent. Jones and Carter (2004) described an increase in the *S*2 and ST intensity domains, whereas Boone et al. (2012) found an increase in the *S*1 and a reduction in the *S*2 domain. Marles et al. (2006) and Ferguson et al. (2007) did not find any change of $\Delta \dot{V}{\text{O}}_{{2}} /\Delta P$ after an intensive prior exercise.

These contradictory findings are potentially caused by methodological differences in terms of the protocol of the prior exercise and the subsequent recovery. The aim of the present study was to test whether the described increase in the $\dot{V}{\text{O}}_{{2}}$-plateau incidence after an intensive prior exercise (Gordon et al. 2012) is caused by a higher $\Delta \dot{V}{\text{O}}_{{2}} /\Delta P$, which results in faster attainment of $\dot{V}{\text{O}}_{{{\text{2max}}}}$ and therefore contributes to the development of a $\dot{V}{\text{O}}_{{2}}$-plateau despite a similar *P*_max_. Thus, we chose a very similar experimental design as described by Gordon et al. (2012). In contrast to our study and the study of Gordon et al. (2012), Jones & Carter (2004), as well as Boone et al. (2012) used incremental ramp tests as prior exercises. Since these ramp tests were performed up to exhaustion, it is likely that the priming interventions were more intensive than in our and the Gordon et al (2012) study. However, the BLC_BSL_ immediately before the start of the primed ramp test in our study was very similar compared to the corresponding values reported by Jones and Carter (2004) and Boone et al. (2012). Furthermore, it has been shown that even a priming exercise in the heavy intensity domain, which goes along with much lower BLC values, led to speeding of $\dot{V}{\text{O}}_{{2}}$ kinetics (Burnley et al. 2000, 2006). Consequently, it seems to be rather unlikely that the intensity of the prior exercise was too low to induce a speeding of $\dot{V}{\text{O}}_{{2}}$ overall kinetics and a corresponding increase in $\Delta \dot{V}{\text{O}}_{{2}} /\Delta P$.

In accordance with the study of Gordon et al. (2012), we chose a 6-min active recovery between the prior exercise bout and the primed ramp test. Thus, the recovery protocol of our study was slightly different from the studies of Jones and Carter (2004) and Boone et al. (2012), which used a 10-min active recovery (Jones and Carter 2004) or a 3-min rest followed by a 3-min active recovery (Boone et al. 2012). It seems to be possible that the duration or kind of recovery (rest vs. low-intensity cycling) affects the $\dot{V}{\text{O}}_{{2}}$-ramp test kinetics.

As shown in Table 2 and Fig. 1, $\dot{V}{\text{O}}_{{2}}$ at baseline-cycling preceding the ramp tests was significantly elevated in the primed condition. A potential explanation for this may be an increased activation of less efficient type 2 muscles fibers (Han et al. 2003). However, this explanation is rather unlikely because the EMG signal did not differ between the primed and the unprimed ramp test. Instead, it seems to be likely that the elevated $\dot{V}{\text{O}}_{{2}}$ at baseline-cycling and the beginning of the ramp test results from an elevated $\dot{V}{\text{O}}_{{2}}$ demand, which is caused by the same mechanisms that are responsible for the excess post-exercise oxygen consumption (EPOC) (Børsheim and Bahr 2003). The EPOC leads to an increase in $\dot{V}{\text{O}}_{{2}}$ not only at rest but also at subsequent low-intensity exercise (Bangsbo et al. 1994; Børsheim and Bahr 2003). The EPOC decreases exponentially with time (Børsheim and Bahr 2003). Therefore, with respect to the rather short recovery duration used in our study, it is possible that a potential increase in $\Delta \dot{V}{\text{O}}_{{2}} /\Delta P$ in the *S*1 and ST domain is superimposed by the EPOC kinetics. Furthermore, the reduction in EPOC with time is much more pronounced during rest compared to active recovery, as described by Bangsbo et al. (1994). This may explain why Boone et al. (2012) found an increase in $\Delta \dot{V}{\text{O}}_{{2}} /\Delta P$ in the *S*1 domain, despite using a recovery duration of 6 min also. Therefore, a passive and/or longer recovery phase may be more suitable to induce an increase in $\Delta \dot{V}{\text{O}}_{{2}} /\Delta P$. However, the effect of the recovery mode or duration on the change of ramp tests kinetics after a priming exercise has never been examined.

At the first glance, the priming-induced reduction in $\Delta \dot{V}{\text{O}}_{{2}} /\Delta P$ in the *S*2 intensity domain seems to be caused by a reduced slow component of $\dot{V}{\text{O}}_{{2}}$-kinetics. Especially in ramp tests with low incremental rates (< 30 W min^−1^), a slightly upward deflection of the $\dot{V}{\text{O}}_{{2}}$–workload-relationship has been reported at workloads above *P*_GET_ (Boone and Bourgois 2012). This upward deflection has been related to the same mechanisms that are responsible for the slow component of $\dot{V}{\text{O}}_{{2}}$-kinetics (Grassi et al. 2015). It is well known that a priming exercise in the heavy or severe intensity domain reduces the magnitude of the slow component (Bailey et al. 2009; Burnley et al. 2000; 2006). However, this reduction is not based on a lower $\dot{V}{\text{O}}_{{2}}$ at the end of a constant load bout, which would indicate a lower gain of overall $\dot{V}{\text{O}}_{{2}}$-kinetics (i.e., a higher delta-efficiency). Instead, the reduction in the slow component is caused by a higher amplitude of the primary/fast component of $\dot{V}{\text{O}}_{{2}}$-kinetics (Bailey et al. 2009; Burnley et al. 2000, 2006). Thus, an increase in the fast component amplitude and a resulting speeding of $\dot{V}{\text{O}}_{{2}}$ overall kinetics should lead to an increase in $\Delta \dot{V}{\text{O}}_{{2}} /\Delta P$ in the *S*2 and ST intensity domains (Wilcox et al. 2016), as demonstrated by Jones and Carter (2004). Reasons for our contrary findings are unclear and must be examined in subsequent studies.

### Effect of prior exercise on ∆RMS/∆P

In the study of Boone et al. (2012), the increase in $\Delta \dot{V}{\text{O}}_{{2}} /\Delta P$ in the S1 intensity domain was accompanied by a steeper increase in the integrated EMG-signal of the left vastus lateralis. This indicates that the higher $\Delta \dot{V}{\text{O}}_{{2}} /\Delta P$ is caused by elevated muscle fiber activation. Despite recording the EMG-signal from VL, VM and GM of each leg, we did not find any changes in ∆RMS/∆P. Since we also did not find an increase in $\Delta \dot{V}{\text{O}}_{{2}} /\Delta P$ in the *S*1 intensity domain, the similar ∆RMS/∆P in the unprimed and primed ramp test does not challenge the finding that the priming-induced increase in $\Delta \dot{V}{\text{O}}_{{2}} /\Delta P$ is caused by elevated muscle fiber recruitment (Boone et al. 2012).

The reasons for these different findings are unclear. Unlike our study, Boone et al. (2012) used a ramp exercise bout as a priming intervention. This approach enables to measure muscle fiber activation of the primed and unprimed ramp test within the same electrode application. However, we tagged the position of the electrodes with an indelible marker to ensure the same position despite the ramp tests being performed on different days. Furthermore, the RMS signal was normalized to the mean RMS of the last minute of the 50 W-baseline cycling. Therefore, it is unlikely that the divergent findings are caused by changes in the electrode applications.

As described above, the recovery protocol of our study was slightly different compared to the study of Boone et al. (2012). Therefore, it cannot be excluded that the absence of an increase in ∆RMS/∆P in our study is caused by the use of a 6-min active recovery instead of a 3-min rest followed by a 3-min baseline-cycling at 50 W, as performed by Boone et al. (2012).

## Conclusions

Contrary to our hypotheses and previous studies, we were not able to demonstrate an increase in plateau incidence, $\Delta \dot{V}{\text{O}}_{{2}} /\Delta P$ and ∆RMS/∆P following an intensive prior exercise. This is potentially caused by differences in the priming exercise or the recovery period between the warm-up and the ramp test, which has to be checked in subsequent studies.

## Abbreviations

BLC: Blood lactate concentration
BLC_BSL_: Blood lactate concentration at 50 W baseline cycling
BLC_max_: Maximal blood lactate concentration
EMG: Electromyography
GM: Gastrocnemius medialis
MRT: Mean response time of ramp test oxygen uptake kinetics
*P*_GET_: Workload at gas exchange threshold
*P*_max_: Maximum workload achieved at ramp test termination
RER_max_: Maximal respiratory exchange ratio
RMS: Root mean square of the electromyography signal
RMS_BSL_: Electromyography root mean square of 50 W baseline cycling
∆RMS/∆P: Slope of the electromyography root mean square–workload-relationship
RPE_max_: Maximal rating of perceived exertion
S: Incremental rate
S1: Intensity between the second minute of the ramp test and *P*_GET_
S2: Intensity between *P*_GET_ and 2 min before ramp test termination
ST: Intensity between the second minute of the ramp test and two minutes before ramp test termination
VL: Vastus lateralis
VM: Vastus medialis
$\dot{V}{\text{CO}}_{{{\text{2max}}}}$: Maximal carbon dioxide production
$$\dot{V}{\text{O}}_{{2}}$$: Oxygen uptake
$\dot{V}{\text{O}}_{{{\text{2BSL}}}}$: Oxygen uptake of 50 W baseline cycling
$\dot{V}{\text{O}}_{{{\text{2max}}}}$: Maximal oxygen uptake
$\Delta \dot{V}{\text{O}}_{{2}} /\Delta P$: Slope of oxygen uptake–workload-relationship
$\Delta \dot{V}{\text{O}}_{{{\text{2max}}}}$: Slope of oxygen uptake–workload-relationship of the final 50 W
